# P-2200. A Retrospective Chart Review of Respiratory Syncytial Virus-related Hospitalizations in Adults

**DOI:** 10.1093/ofid/ofaf695.2363

**Published:** 2026-01-11

**Authors:** Stephen Baker, Abdullah Salama, Luke Shea, Ross Bernstein, Katherine S Getman, Quinlan Z Wu, Shane J Sacco, Jessica Abrantes-Figueiredo, Eun Sun Lee, Kevin Dieckhaus

**Affiliations:** University of Connecticut, West Hartford, CT; University of Connecticut School of Medicine, Avon, Connecticut; 3UConn SOM, Farmington, Connecticut; University of Connecticut School of Medicine, Avon, Connecticut; UConn, Wolcott, Connecticut; Boston University, Unionville, Connecticut; UConn Health Center, Farmington, Connecticut; Saint Francis Hospital, Hartford, Connecticut; Hartford Hospital, Hartford, Connecticut; UConn Health, Southington, Connecticut

## Abstract

**Background:**

Respiratory syncytial virus (RSV) causes annual outbreaks of respiratory illness with peak incidence occurring from late fall to early spring. While RSV is a leading cause of pediatric hospitalizations, it also poses a significant burden on older adults.

**Table 1.**
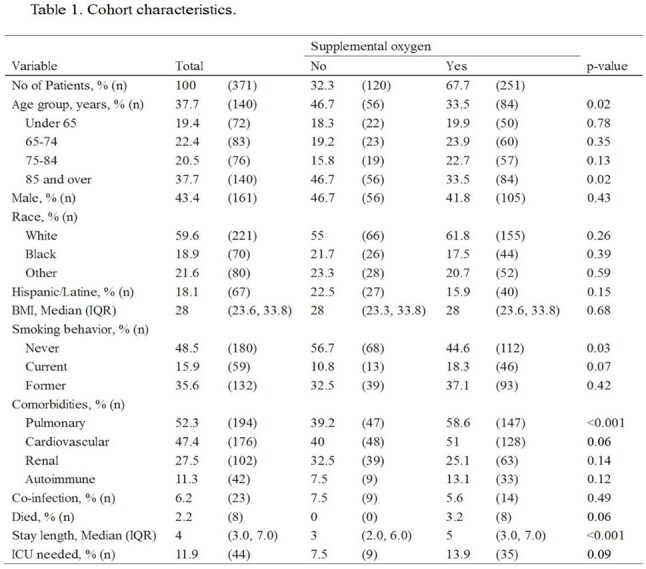
Cohort Characteristics

**Table 2.**
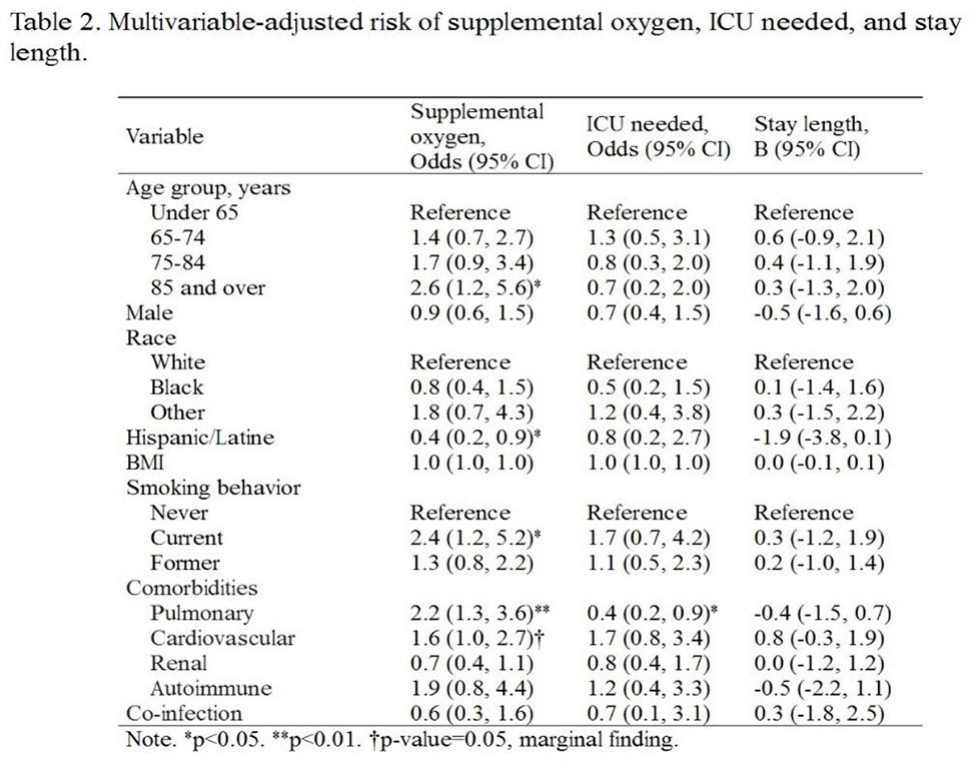
Multivariable-adjusted risk of supplemental oxygen, ICU needed, and stay length

**Methods:**

We reviewed electronic medical records for hospitalized adults with laboratory-confirmed RSV between July 1, 2020, and June 30, 2024. Demographics, risk factors and outcomes were recorded. We created logistic and linear regression models to obtain multivariable-adjusted risks of supplemental oxygen needs, ICU level care, and increased length of stay (LOS).

**Results:**

371 patients met inclusion criteria. The cohort was predominantly older (≥65 years, 62%), White (60%), and female (57%), and were also in generally poor health (e.g., overweight BMIs, ∼50% current or former smokers, ∼50% with pulmonary or cardiovascular disease). Around one third required supplemental oxygen, 12% required ICU care and 2% died. The median LOS was 4 days, and both needing oxygen and ICU care associated with longer LOS (ps< 0.001). Multivariable-adjusted models suggested patients 85 years or older were at 160% higher risk to need supplemental oxygen; current smokers were at 140% higher risk, pulmonary patients 120%, and cardiac patients 70% (ps≤0.05); Hispanic or Latine patients were 60% at lower risk (p=0.04). Patients with pulmonary disease history had a 60% lower risk of needing ICU care (p=0.02). No demographic variables predicted LOS (ps >0.06).

**Conclusion:**

We identified key demographic and clinical characteristics associated with greater illness severity of RSV, including the need for supplemental oxygen and ICU care. The roughly 1 in 3 requirements for supplemental oxygen, 12% ICU admission rate, and 2% in-hospital mortality highlight the considerable burden RSV imposes on hospitalized adults. Our findings suggest that elderly adults and those with underlying cardiopulmonary disease represent particularly high-risk groups for severe RSV manifestations. Current smokers and those over age 85 each had over twice the odds of requiring supplemental oxygen compared to younger patients and never-smokers. Protocols for RSV prevention—such as vaccination, early detection, and respiratory support may help optimize hospital outcomes.

**Disclosures:**

All Authors: No reported disclosures

